# Comparative efficacy and safety of different drugs for bipolar disorder complicated with anxiety disorder: A protocol for systematic review and network meta-analysis

**DOI:** 10.1097/MD.0000000000022280

**Published:** 2020-09-18

**Authors:** Li Yang, Meili Yan, Li Du, Shasha Hu, Zhigang Zhang

**Affiliations:** a Intensive-Care Unit, The First People's Hospital of Lanzhou City, Lanzhou, China; b Evidence-Based Nursing Center, School of Nursing, Lanzhou University, Lanzhou, China; c Psychiatry Department, The Third People's Hospital of Lanzhou City, Lanzhou, China; d Gynecology Department, The First Hospital of Lanzhou University, Lanzhou, China; e Intensive-Care Unit, The First Hospital of Lanzhou University, Lanzhou, China.

**Keywords:** adverse effect, anxiety disorder, bipolar disorder, efficacy, network meta-analysis

## Abstract

**Background:**

Nowadays, there are some randomized controlled trials (RCTs) to explore the effectiveness of drug therapy for bipolar disorder with anxiety disorders. However, due to lack of sufficient data, there are currently no good treatment recommendations. The purpose of this network meta-analysis is to compare the efficacy and safety of different drugs for bipolar disorder complicated with anxiety disorders to provide evidence to support clinical practice and guidelines development.

**Methods:**

A systematic literature search will be performed in the Cochrane Library, PubMed, EMBASE, and Web of Science from inception to July 2020. RCTs that compared the efficacy and safety of different drugs for bipolar disorder complicated with anxiety disorders will be included. Two reviewers will independently search and select the studies, extract the data, and assess the risk of bias. We will assess the risk of bias of included RCTs using the Cochrane risk of bias tool. The WinBUGS 1.4.3 software will be used to perform the network meta-analysis, and the result figures will be generated by STATA 15.0 software. In addition, we will use the Grading of Recommendations Assessment, Development and Evaluation (GRADE) to assess the quality of evidence.

**Results:**

This study will systematically compare the efficacy and safety of different drugs for bipolar disorder complicated with anxiety disorders. The results of this network meta-analysis will be submitted to a peer-reviewed journal for publication.

**Conclusion:**

Our study will provide evidence for the drug therapy of patients with bipolar disorder complicated with anxiety disorders, and provide suggestions for clinical practice or guidelines.

**INPLASY registration number:**

INPLASY202070132.

## 1 Introduction

Bipolar disorder (BD) is a chronic and debilitating psychiatric illness with both manic and depressive episodes. It is a disabling mental illness with high morbidity and mortality.^[1]^ And it has a very heavy burden of illness, according to incomplete statistics, the per capita annual cost of BD in the world ranges from $1904 to $33090.^[2]^ If the patients have comorbidities, then the related costs will be higher. Patients diagnosed with BD usually suffer from one or more psychiatric diagnoses, of which anxiety disorders are relatively common. According to statistics, 24% to 56% of patients with BD meet the criteria of one or more anxiety disorders.^[3,4]^ Anxiety disorder is the most prevalent psychiatric disorder; it refers to a constant state of nervousness or episodic panic.^[5]^ Compared with patients without comorbid anxiety disorders, patients with BD comorbid anxiety disorders have an earlier age of illness onset and generally have more severe symptoms,^[6]^ higher incidence of mixed states, depressive symptoms, suicidal ideation and, other psychosocial disorders.^[7]^

Although there are treatment options,^[4]^ the limitations caused by the lack of data have hindered the development of clear treatment suggestions in the guidelines. Drug therapy plays an important role in the treatment of BD with anxiety disorders.^[8]^ Several randomized controlled trials (RCTs) have explored the effects of drug therapy for BD comorbid anxiety disorders, but there are a wide variety of drugs involved and the quality of RCTs is also jagged. To better provide evidence for the practice of evidence-based medicine,^[9]^ we conducted a network meta-analysis (NMA) to screen out the best evidence of drug treatment.^[10]^

NMA is an extension of traditional meta-analysis, which can compare the efficacy of 3 or more interventions at the same time.^[11]^ It allows comparisons of more than 2 interventions in a single, coherent analysis of all the relevant RCTs when multiple studies are available; it can also be used to combine multiple therapeutic effects and obtain an overall estimate of the effects in the target population. The main advantage is that it can quantitatively compare different measures for the treatment of similar diseases, and rank interventions according to the effect of a certain result index, and then choose the best treatment plan.^[12,13]^

The purpose of this systematic review and NMA is to compare the efficacy and safety of drugs in the treatment of patients with BD complicated with anxiety disorders, to screen out the best drug to provide a better basis for clinical practice and psychological services based on health policies.

## 2 Methods

### 2.1 Eligibility criteria

#### 2.1.1 Type of study

RCTs that compared the efficacy and safety of different drugs for the treatment of patients with BD complicated with anxiety disorders will be included. We will include RCTs written in the English language.

#### 2.1.2 Type of patients

Patients with BD complicated with anxiety disorders who meet the following criteria: aged 18 to 65 years, bipolar I disorder, bipolar II disorder, or NOS (nonspecific BD) diagnosed by DSM-IV criteria, confirmed by the Structured Clinical Interview for DSM-IV-Patient Edition (SCID-I/P)^[14]^; and anxiety disorders diagnosed by DSM-V criteria [it includes separation anxiety disorder, selective mutism, specific phobia, social anxiety disorder, panic disorder, agoraphobia, generalized anxiety disorder (GAD), and panic attack specifier].^[15]^

#### 2.1.3 Type of interventions

We will include all published and unpublished RCTs that reported different drug treatments in patients with BD complicated with anxiety disorders. The intervention group includes any pharmacological treatments, such as olanzapine, risperidone, and ziprasidone. The controlled group is a placebo or any active drugs that are used in clinical practice.

#### 2.1.4 Type of outcomes

The main outcome measure is the efficacy, measured by the following anxiety symptom scales of the overall mean change scores between baseline and week 8 (range 4–12 weeks).

(1) overall mean change scores on the Hamilton Anxiety Scale (HAM-A);
(2) overall mean change scores on the Clinician Global Improvement Scale for Anxiety (CGI-21 Anxiety);
(3) overall mean change scores on the Sheehan Panic Disorder Scale (SPS).

Safety is the secondary outcome; we mainly measure it through the rating scale of adverse drug reactions, which is commonly used in the psychiatric department in clinical practice, including the Rating Scale for Extrapyramidal Side Effects (RSESE) and the Udvalg for the Kliniske Undersogelser (UKU).

### 2.2 Data source and search strategy

Electronic databases will be searched from inception to July 30, 2020. Databases searched include the Cochrane Library, PubMed, EMBASE, and Web of Science. The search strategy will be adapted to each database; the search terms include “bipolar disorder,” “bipolar affective disorder,” “manic depressive,” “manic depressive psychosis,” “anxiety disorder,” and others (for the full search strategy, please see Tables 1 and 2). We will also search major trial registries for ongoing trials, including the Cochrane Central Register of Controlled Trials (CENTRAL), the WHO International Clinical Trials Registry Platform (WHO ICTRP), the International Standard Randomized Controlled Trial Number (ISRCTN) Registry, and ClinicalTrials. Furthermore, reference lists of included RCTs and relevant systematic reviews will be searched. There will be no restrictions on publication year.

**Table 1:**
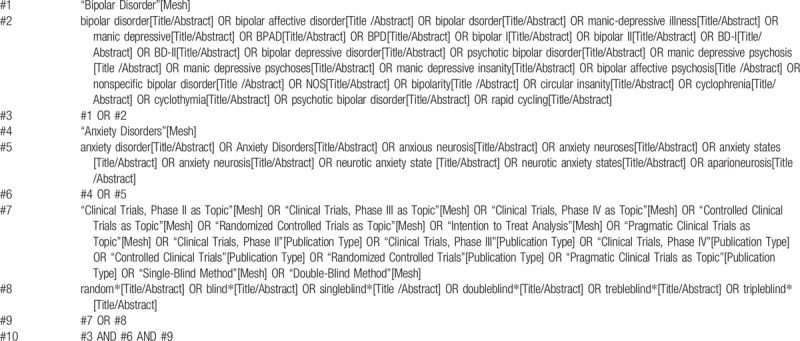
Searching strategy in PubMed.

**Table 2:**
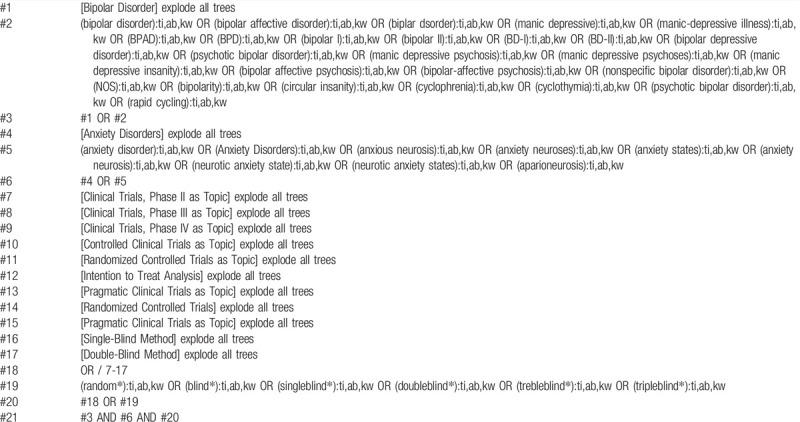
Searching strategy in the Cochrane Library.

### 2.3 Study selection

Literature search records will be imported into ENDNOTE X8 literature management software; we will use Microsoft Excel 2013 to create a standard data extraction form to collect relevant information and data, which include the name of first author, year of publication, the country in which the study was conducted, the sample size, interventions, implementation of blinding, outcomes, patient's characteristics (gender, age, the severity of illness, course, duration) and adverse reactions, etc.

### 2.4 Data extraction

We will use Microsoft Excel 2013 to create a standard data extraction form to collect relevant information and data, which include the name of first author, year of publication, the country in which the study was conducted, the sample size, interventions, implementation of blinding, outcomes, patient's characteristics (gender, age, the severity of illness, course, duration), and adverse reactions. The data will be extracted independently by 2 reviewers using standardized data extraction forms. Any disagreements will be resolved through discussion between the 2 parties or decided by a more qualified third party.

### 2.5 Risk of bias of individual studies

The methodological quality of the included RCTs will be independently evaluated by 2 reviewers. Any disagreements will be resolved through discussion between the two parties or decided by a third reviewer. Consistent with the Cochrane Handbook for Systematic Reviews of Interventions,^[16]^ we will use the Cochrane bias risk assessment tool to evaluate the risk of bias; the following items will be evaluated: ① random sequence generation, ② allocation sequence concealment, ③ blinding of participants and personnel, ④ blinding of outcome assessment, ⑤ incomplete outcome data, ⑥ selective outcome reporting, and ⑦ other biases.

### 2.6 Statistical analysis

#### 2.6.1 Network meta-analysis

First of all, we will use the random effect model of Stata 15.0 to conduct a paired meta-analysis of direct evidence. We will calculate the mean differences (MDs) or standardized mean differences (SMDs) with 95% confidence interval (95% CI) for continuous variable data, and relative risk (RR) with 95% CI for dichotomous variable data. The statistical heterogeneity will be examined using the *I*^2^ statistic and *P* value. If the *P* value ≥.1 or *I*^2^ ≤50%, it suggests that there is no statistical heterogeneity; if not, we will explore sources of heterogeneity by subgroup analysis and meta-regression. If applicable, Egger test and funnel plot will be used to evaluate the potential publication bias.^[17,18]^ Second, we will use the Markov chain Monte Carlo method in WinBUGS V.1.4.3 (MRC Biostatistics Unit, Cambridge, UK) to perform NMA with the random-effects model in the Bayesian framework.^[19]^ We will use the node splitting method to examine the inconsistency between direct and indirect comparisons. Besides, we will use the surface under the cumulative ranking curve (SUCRA) for the treatment of patients with BD complicated with anxiety disorders, and the ranking probability of the efficacy and safety of different drugs will be estimated. The results of the rankograms, ranking probabilities plots, and evidence network graph will also be presented graphically.^[20]^

#### 2.6.2 Subgroup analysis and sensitivity analysis

Where possible, we will conduct the network meta-regression analyses of data on primary outcomes for the age of participants; sex; the severity of BD symptoms at baseline; the severity of anxiety symptoms at baseline; and the treatment duration. If possible, we will do some extra subgroup analyses according to the results of heterogeneity and inconsistency. If the evidence is sufficient, we will conduct the sensitivity analysis by excluding trials with imputed missing data, trials with a high risk of bias, and trials that only included patients comorbidity with other psychiatric disorders.^[21]^ We will also investigate the sources of heterogeneity to determine the robustness and reliability of the consolidated results.

### 2.7 Quality of evidence and summary of findings

We will use the Grading of Recommendations Assessment, Development and Evaluation (GRADE) to assess the quality of evidence; these 5 considerations (study limitations, consistency of effect, imprecision, indirectness, and publication bias) will be applied to assess the quality of evidence.^[22,23]^ The certainty of evidence will be categorized into 4 levels: high, moderate, low, and very low.

We will create a “summary of findings” table for the major outcomes as well as add the absolute and relative percent change in the “summary of findings” table, for details, please see Table 3; we have listed a partial summary of findings for the main comparisons.

**Table 3:**
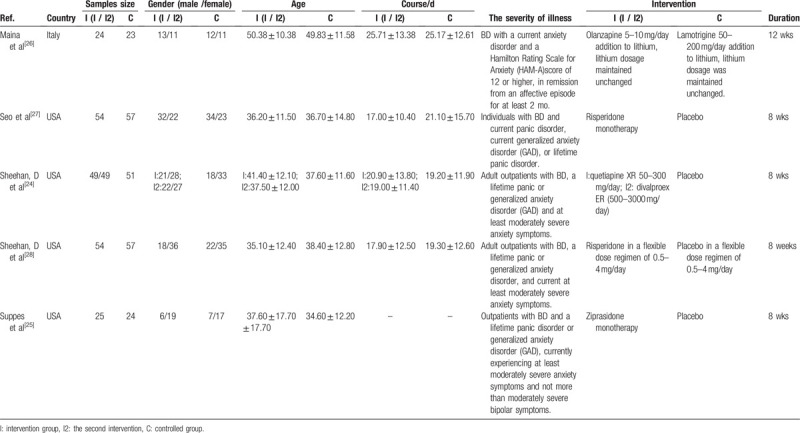
Characteristics of some of the included studies.

## 3 Results

### 3.1 Results of the search

**Figure 1:**
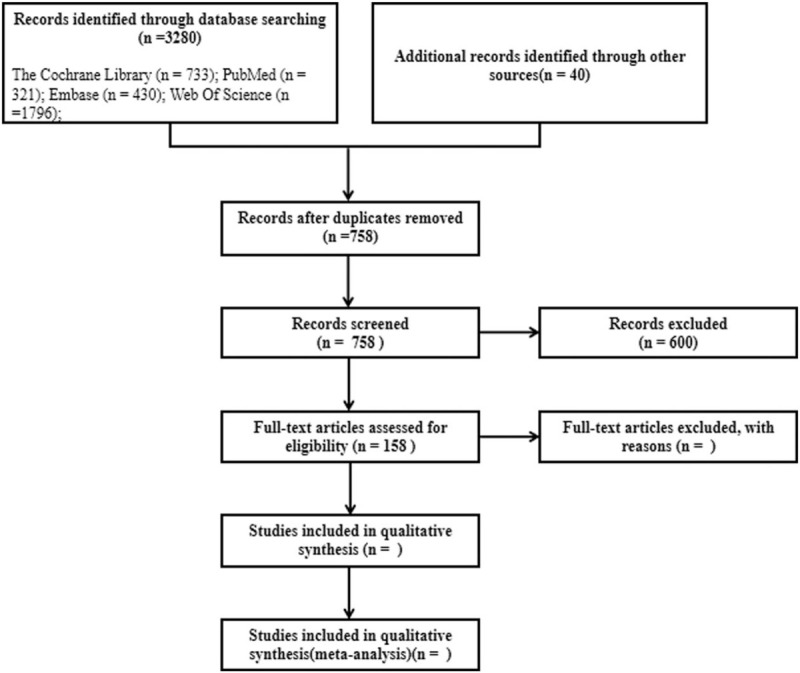
Flowchart for the selection of eligible studies.

We identified 3280 records through database searching and 40 records through other sources. After eliminating duplicates, we screened 758 records. We excluded 600 records after reviewing their titles and abstracts, leaving 158 full-text articles. The detailed search flowchart is shown in Figure 1.

### 3.2 Characteristics of included studies

We conducted a preliminary experiment and included 5 RCTs; the minimum sample size is 47 and the maximum is 149. The mean age ranged from 35 to 51 years, and the course of disease ranged from 8 to 12 weeks. For further details, please refer to the characteristics of some of the included studies (Table 4).^[24–28]^

**Table 4:**
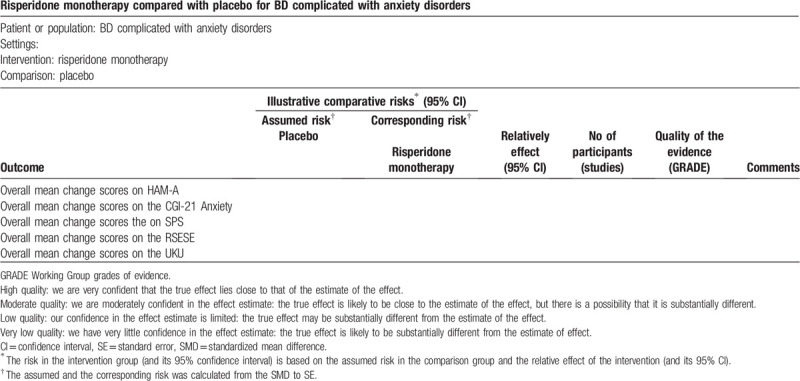
Partial summary of findings for the main comparisons.

## 4 Discussion

To the best of our knowledge, this study will be the first NMA comparing the efficacy and safety of different drugs in the treatment of BD complicated with anxiety disorders. This NMA will summarize the direct and indirect evidence to compare the efficacy and safety of different drugs in the treatment of this comorbidity. The design of the protocol follows the guidelines of the NMA protocol, and the NMA will be carried out in accordance with the PRISMA extension statement to implement and report this protocol strictly.^[29,30]^

In addition, we will use the GRADE framework to assess the quality of the evidence. We hope that this study could screen out the best drugs to provide evidence of drug treatment for patients with BD complicated with anxiety disorders and provide suggestions for clinical practice or guidelines.

## Acknowledgments

We would like to thank their valuable advice and assistance of Dr. Jinhui Tian, Dr. Kelu Yang, Dr. Ya Gao, Dr. Yitong Cai, Dr. Jiyuan Shi, Dr. Pengzhong Fang on the methodology and quality of our study.

## Author contributions

Author 1 - Li Yang - Provided methodological advice, polished, and revised the manuscript.

Author 2 - Meili Yan - Study design, data extraction, and drafted the manuscript.

Author 3 - Li Du - In charge of extracting data and verification, and provided expertise on treatments, outcomes, and related knowledge.

Author 4 - Shasha Hu - was the corresponding author, responsible for all work of the review.

Author 5 - Zhigang Zhang - was the corresponding author and approved the final version of the manuscript.

**Data curation:** Li Du.

**Methodology:** Li Yang.

**Supervision:** Shasha Hu.

**Writing – original draft:** Meili Yan.

**Writing – review & editing:** Zhigang Zhang.

## Abbreviations

Abbreviations: BD = bipolar disorder, CGI-21 Anxiety = the Clinician Global Improvement - 21 Anxiety Scale, GAD = generalized anxiety disorder, GRADE = Grading of Recommendations Assessment, Development and Evaluation, HAM-A = Hamilton Anxiety Scale, NMA = network meta-analysis, NOS = nonspecific bipolar disorder, PICOS = participants, interventions, comparisons, outcomes and study design, RCT = randomized controlled trial, RSESE = Rating Scale for Extrapyramidal Side Effects, SMD = standard mean difference, SPS = Sheehan Panic Disorder Scale, UKU = Udvalg for Kliniske Undersogelser.
